# Chemical-free and synergistic interaction of ultrasound combined with plasma-activated water (PAW) to enhance microbial inactivation in chicken meat and skin

**DOI:** 10.1038/s41598-020-58199-w

**Published:** 2020-01-31

**Authors:** Tanitta Royintarat, Eun Ha Choi, Dheerawan Boonyawan, Phisit Seesuriyachan, Wassanai Wattanutchariya

**Affiliations:** 1 Advanced Manufacturing Technology Research Center (AMTech), Department of Industrial Engineering, Faculty of Engineering, Chiang Mai University, Chiang Mai, 50200 Thailand; 2 Plasma Bioscience Research Center, Kwangwoon University, Seoul, Korea; 3 Department of Physics and Materials Science, Faculty of Science, Chiang Mai University, Chiang Mai, 50200 Thailand; 4 Cluster of Agro Bio-Circular-Green Industry, Faculty of Agro-Industry, Chiang Mai University, Chiang Mai, 50100 Thailand

**Keywords:** Industrial microbiology, Plasma physics

## Abstract

In general, the poultry industry uses 0.5–1 ppm chlorine solution in the meat sanitization process. However, chlorine can react with organic material and produce halogenated organic compounds, notably chloroform, which causes bladder and rectal cancer in humans. For this reason, many industries try to avoid chlorine. This study investigated the efficacy of ultrasound and plasma-activated water (PAW) on the inactivation of *Escherichia coli* and *Staphylococcus aureus* in chicken muscle, rough skin, and smooth skin. Samples inoculated with bacteria suspension were treated by ultrasound alone and PAW–ultrasound. The Taguchi method and desirability function approach were used for the experimental design and optimization. Combined ultrasound and PAW inactivated up to 1.33 log CFU/ml of *E. coli* K12 and 0.83 log CFU/ml of *S. aureus* at a sample thickness of 4 mm, at 40 °C for 60 min, while PAW alone only reduced *E. coli* K12 by 0.46 log CFU/ml and *S. aureus* by 0.33 log CFU/ml under the same condition. The muscle topography showed a porous structure, which facilitated the penetration of PAW. The color measurements of muscle treated with ultrasound and PAW–ultrasound were dramatically different from the untreated sample, as also perceived by the sensory evaluation panel. Therefore, the synergistic interaction of combined PAW–ultrasound could be used to enhance microbial inactivation in meat.

## Introduction

In the past, sanitization by the chilling process with circulation system in the poultry industry used ice and 0.5–1 ppm chlorine to decrease the temperature of chicken carcass and lower the bacterial load in the gizzard and intestine. However, this process can cause cross-contamination because of circulated poultry water in the chiller tank. Additionally, chlorine can react with organic materials and produce halogenated organic compounds, notably, chloroform, which causes bladder and rectal cancer in humans.

Various technologies have since been developed to reduce the bacteria in chicken meat, such as bacteriophage (ListShield™) combined with UV-C light reduced *Listeria monocytogenes* 2.04 log CFU/ml^1^, ultrasound combined with a chemical immersion (lactic acid, sodium decanoate, and trisodium phosphate) reduced 0.73 log CFU/ml of *Campylobacter jejuni*, 1.02 log CFU/ml of TVC, and 1.37 log CFU/ml of total Enterobacteriaceae^2^, electrolyzed water reduced 1.0 log CFU/ml of microbial reduction^3^, non-thermal plasma jet with N_2_/O_2_ gas reduced 0.66 log CFU/g of *Salmonella typhimurium*^4^, cold atmospheric plasma pen with He/O_2_ gas reduced 3 log CFU/ml of *L. innocua* on muscle^5^, and dielectric barrier discharge plasma reduced 3.11 log CFU/ml of *C. jejuni* on skin^6^.

The past decade has focused on an environmentally-clean sanitizing technology called non-thermal plasma, particularly plasma-activated water (PAW). PAW can be used as a bacterial disinfection reagent in mass production because of low operation cost compared with other technologies such as chlorination, ozonation, electrochemical processes^7^ and thermal plasma^8^ without changing the product characteristics. The main chemical components that affect bacteria are reactive oxygen species (ROS) and reactive nitrogen species (RNS), together called reactive oxygen and nitrogen species (RONS). The RONS consist of the hydroxyl radical (•OH), hydrogen peroxide (H_2_O_2_), the superoxide radical (•O_2_^−^), oxygen (O_2_), and the nitric oxide radical (•NO). These species damage the DNA inside of bacteria. Therefore, cross-contamination is reduced. The main chemical reactions of RONS generated by plasma treatment of water can be explained by Eqs. (1)–(8):

1 $${{\rm{H}}}_{2}{\rm{O}}\to \bullet {\rm{OH}}+\bullet {\rm{H}}$$

2 $$\bullet {\rm{OH}}+\bullet {\rm{OH}}\to {{\rm{H}}}_{2}{{\rm{O}}}_{2}$$

3 $$\bullet {\rm{H}}+\bullet {\rm{H}}\to {{\rm{H}}}_{2}$$

4 $${{\rm{O}}}_{2}\to {{\rm{O}}}^{\mbox{--}}+{\rm{O}}$$

5 $${\rm{O}}+{{\rm{O}}}_{2}\to {{\rm{O}}}_{3}$$

6 $${{\rm{N}}}_{2}\to \bullet {\rm{N}}+\bullet {\rm{N}}$$

7 $${{\rm{O}}}_{2}^{\mbox{--}}+\bullet {\rm{N}}\to \bullet {\rm{NO}}$$

8 $${{\rm{O}}}_{2}+\bullet {\rm{H}}\to \bullet {{\rm{HO}}}_{2}^{\mbox{--}}\leftrightarrow {{\rm{H}}}^{+}+{{\rm{O}}}_{2}^{\mbox{--}}$$

Another low-temperature technique that has been used to inactivate bacteria is ultrasound. The advantage of ultrasound, besides the decrease in microbial load, is the increase in the water holding capacity of meat, preventing marinate and water losses in fresh meat^9^. The cavitation bubbles generated by ultrasound can have varying effects within the liquid medium, depending on whether the system is a homogenous liquid, heterogeneous solid/liquid, or heterogenous liquid/liquid type^10^. In the homogeneous liquid phase system, the collapse of the cavitation bubbles generates extreme high-temperature and pressure conditions at low-frequency ultrasound (16–100 kHz), which produces H atoms and •OH radicals^11^. Moreover, DNA damage occurs at high temperature, due to the ultrasonic intensity above the cavitation threshold can generate RONS^12^. The accepted inactivation mechanisms of ultrasound are acoustic cavitation, micromechanical shockwaves, compression and rarefaction, and sonochemical reactions^13^. The RONS chemical reactions triggered by ultrasound-induced decomposition of water are described by Eqs. (9)–(14). The pyrolysis reactions in Eqs. (12) and (13) differentiate ultrasound-induced reactions from those that originate from plasma treatment.

9 $${{\rm{H}}}_{2}{\rm{O}}\to \bullet {\rm{OH}}+\bullet {\rm{H}}$$

10 $$\bullet {\rm{OH}}+\bullet {\rm{OH}}\to {{\rm{H}}}_{2}{{\rm{O}}}_{2}$$

11 $$\bullet {\rm{H}}+\bullet {\rm{H}}\to {{\rm{H}}}_{2}$$

12 $$\bullet {\rm{OH}}+\bullet {\rm{OH}}\to {{\rm{O}}}_{2}^{\mbox{--}}+{{\rm{H}}}_{2}{\rm{O}}$$

13 $${{\rm{O}}}_{2}^{-}+{{\rm{N}}}_{2}\to \bullet {\rm{NO}}+\bullet {\rm{N}}$$

14 $${{\rm{O}}}_{2}+\bullet {\rm{H}}\to \bullet {{\rm{HO}}}_{2}^{\mbox{--}}\leftrightarrow {\rm{H}}++{{\rm{O}}}_{2}^{\mbox{--}}$$

The ultrasound involves heterogeneous solid surface-liquid systems, which show a different behavior from cavitation bubble collapse in bulk liquid^14^. The behavior is like a liquid jet, targeted at the surface of the solid in the liquid system. The interfacial boundary layers are disrupted by the heat and mass transfer effects at the surface of the solid^15^. Low-temperature (59 °C) and low-pressure (400 kPa) ultrasound can reduce 5 log CFU/ml of *Escherichia coli* K12 in apple cider^16^, highlighting the inactivation efficacy of ultrasound treatment on bacteria.

Both PAW and ultrasound are considered as environmentally-friendly and cost-effective techniques. Royintarat (2019) has been proved that arc plasma discharge combined ultrasonic can reduce bacteria on chicken meat part^17^ Therefore, this research focused on using PAW from underwater jet and ultrasound to increase the efficiency of bacterial reduction in different types of chicken meat and skin. The research also investigated the pH value, electrical conductivity (EC) value, oxidation–reduction potential (ORP) value, and •OH and H_2_O_2_ concentrations generated by PAW and ultrasound, which could play a significant role in the antimicrobial mechanism. This combined technology approach could be employed for bacterial inactivation in the poultry industry to replace the chlorine sanitization process. Moreover, this study will focus on using the desirability function approach (DFA) to analyze the relationship between variable factors and responses of PAW towards bacteria reduction in chicken meat and skin.

## Results

### Physicochemical properties of PAW and ultrasound

**Figure 1:**
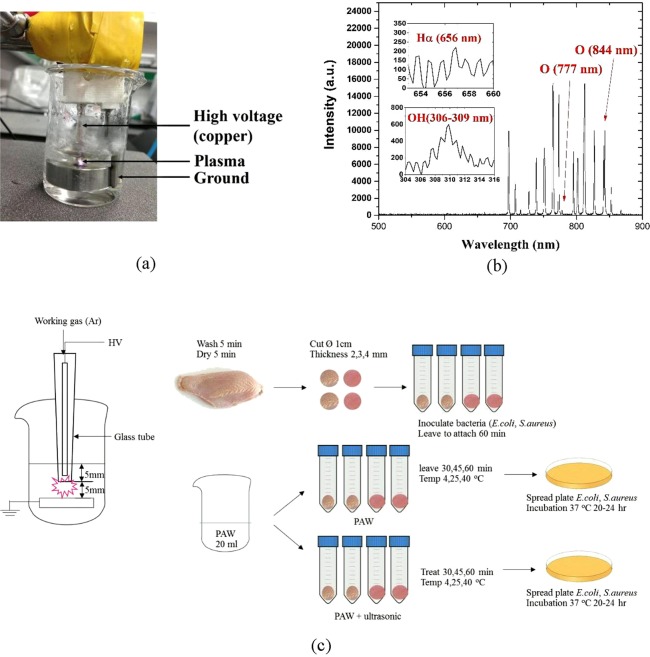
(**a**) A photograph of PAW generation (**b**) optical emission spectra of Ar plasma discharge underwater to generate PAW, created OH (306–309 nm), O (777 nm), and O (844 nm) (**c**) an experimental schematic diagram of PAW and PAW-ultrasound treated chicken sample.

As shown in Fig. 1b, Optimal Emission Spectroscopy (OES) was used to identify the ROS generated by the non-thermal atmospheric plasma. The spectrum was measured at 800 ms. The OES result showed •OH (309 nm), Hα (656 nm), O_777_ (777 nm), and O (844 nm) peaks, which are considered as important ROS of PAW to inactivate bacteria under the optimal condition of the underwater plasma jet.

**Figure 2:**
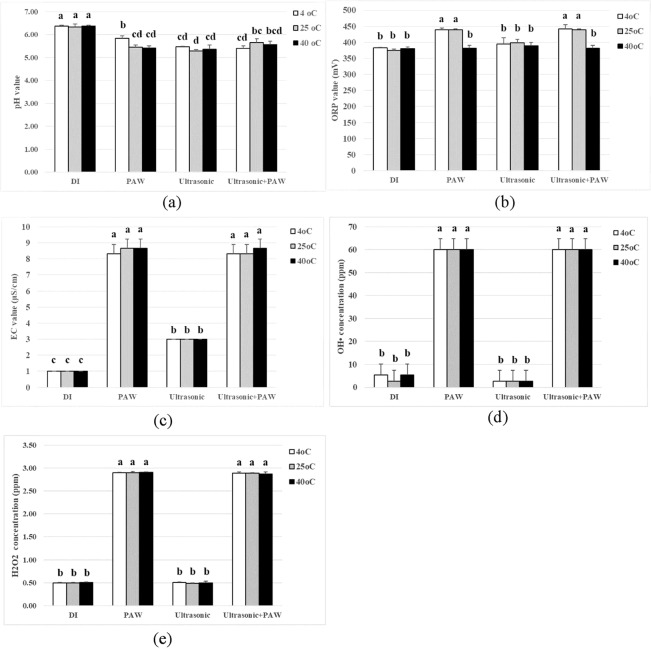
The physicochemical properties of DI water, PAW, ultrasound, and PAW-ultrasound at □ 4 °C,  25 °C and ■ 40 °C (**a**) pH value (**b**) ORP value (mV) (**c**) EC value (μS/cm) (**d**) •OH concentration (ppm) (**e**) H_2_O_2_ concentration (ppm).

The experiment compared the pH, EC, ORP, •OH, and H_2_O_2_ values at 4, 25, and 40 °C using ANOVA and Turkey’s test at *p = *0.05. Figure 2a reveals that the samples treated by PAW, ultrasound, and PAW–ultrasound had a significantly lower pH value when compared with the untreated sample. An acidic change after PAW–ultrasound treatment is mainly derived from •HO_2_ [*pK*_*a*_(•HO_2_/•O^2−^) = 4.8]^18^.

Figure 2b indicates that the ORP value of PAW increased for DI water at 4 and 25 °C, but was stable at 40 °C. At high temperature, the chemical reactions could reduce the oxidation potential of PAW. Shen *et al*.^19^ investigated the physicochemical properties of PAW stored at various temperature (−80, −20, 4, and 25 °C) and noticed that the ORP value was lower when the storage temperature was higher, indicating an inverse association between the temperature and ORP value, according to the Nernst equation.

The EC value demonstrated that the concentration of ions increased from 1 to around 8 μS/cm after treatment by non-thermal plasma. The ultrasound treatment increased the EC of DI water, but not for PAW. However, the temperature change was not affected by the EC value (Fig. 2c). The efficiency of the EC value for killing bacteria can be explained by a physical mechanism called electrostatic disruption^20^. The electrostatic disruption from the accumulation of charged particles disrupts the cell membrane of bacteria when the electric force overcomes the tensile strength.

Figure 2(d,e) display the increasing •OH and H_2_O_2_ concentrations of PAW. However, ultrasound had no significant differences in the •OH and H_2_O_2_ concentrations among the temperatures 4, 25, and 40 °C. In general, the collapse of large cavitation bubbles generated under ultrasound produces extremely high-temperature and pressure in the cavitation zones, generating H atoms and •OH radicals^11^. The experiment shows that the ultrasonic condition is below the threshold, so the H atoms and •OH radicals did not appear.

## Desirability Function Approach (DFA)

The optimum level of parameters satisfied the maximal reduction of *E. coli* and *S. aureus*. All five steps of the optimization process are presented below:

**Step 1:** The results for chicken muscle, rough skin (Table 1), and smooth skin (Table 2) were calculated by Eq. (27) to normalize the value in the range 0–1. Then, these normalized values were raised to the power of 0.5.

**Table 1:** Response table for the composite desirability of each chicken type with PAW and PAW- ultrasound.

Process parameter	Average composite desirability									
	PAW					PAW-ultrasound				
	level 1	level 2	level 3	Delta	Rank	level 1	level 2	level 3	Delta	Rank
**Muscle**										
temp	0.2856	0.3008	0.7896^*^	0.5040	1	0.2102	0.4641	0.6045^*^	0.3943	1
time	0.3765	0.4568	0.5426^*^	0.1661	3	0.2978	0.4296	0.5514^*^	0.2536	3
thickness	0.2080	0.4759	0.6921^*^	0.4842	2	0.2833	0.3963	0.5993^*^	0.3160	2
**Rough skin**										
temp	0.4433	0.6554	0.6993^*^	0.2560	2	0.412	0.6375	0.7393^*^	0.3272	2
time	0.5052	0.5983	0.6945^*^	0.1894	3	0.5065	0.5828	0.6996^*^	0.1931	3
thickness	0.2224	0.6584	0.9171^*^	0.6947	1	0.2626	0.6455	0.8807^*^	0.6181	1
**Smooth skin**										
temp	0.0000	0.9090^*^		0.9092	1	0.0000	0.9264		0.9264	1
time	0.4092	0.5000^*^		0.0908	2	0.4264	0.5000		0.0736	2

*Maximum level of each parameter.

**Table 2:** The analysis of variance (ANOVA) of each chicken type with PAW and PAW- ultrasound.

Muscle	PAW					PAW - ultrasound				
Source	DF	Seq SS	Adj MS	P	%C	DF	Seq SS	Adj MS	P	%C
Temp	2	0.4932	0.2466	0.057	54%	2	0.2397	0.1198	0.15	45%
Time	2	0.0414	0.0207	0.418	5%	2	0.0965	0.0483	0.305	18%
Thickness	2	0.3529	0.1764	0.078	38%	2	0.1538	0.0769	0.216	29%
Error	2	0.0298	0.0149		3%	2	0.0424	0.0212		8%
Total	8	0.9174			100%	8	0.5323			100%
**Rough skin**										
Temp	2	0.1125	0.1125	0.172	12%	2	0.1683	0.0841	0.105	20%
Time	2	0.0538	0.0538	0.302	6%	2	0.0568	0.0284	0.258	7%
Thickness	2	0.7397	0.7397	**0.031**	80%	2	0.5840	0.2920	**0.033**	70%
Error	2	0.0233	0.0233		3%	2	0.0198	0.0099		2%
Total	8	0.9292			100%	8	0.8288			100%
**Smooth skin**										
Temp	1	0.8266	0.8266	0.063	98%	1	0.8582	0.8582	0.05	99%
Time	1	0.0083	0.0083	0.5	1%	1	0.0054	0.0054	0.5	1%
Error	1	0.0083	0.0083		1%	1	0.0054	0.0050		1%
Total	3	0.8431			100%	3	0.8691			100%

**Step 2:** The weighted *d*_*i*_ values were used in Eq. 28 to obtain the *d*_*G*_, which was optimized in the next step. The total mean value of the *d*_*G*_ was compared with the predicted *d*_*G*_ in the final step. For chicken muscle, PAW = 0.4587 and PAW–ultrasound = 0.4263. The rough chicken skin of PAW = 0.5993 and PAW–ultrasound = 0.5963. The smooth, thin chicken skin of PAW = 0.4546 and PAW–ultrasound = 0.4632.

**Step 3:** The optimum condition was determined by considering the maximum level of each parameter. The maximum level will be the best factor, as shown in Table 1. Then, all best factors are combined to give an optimal condition.

**Figure 3:**
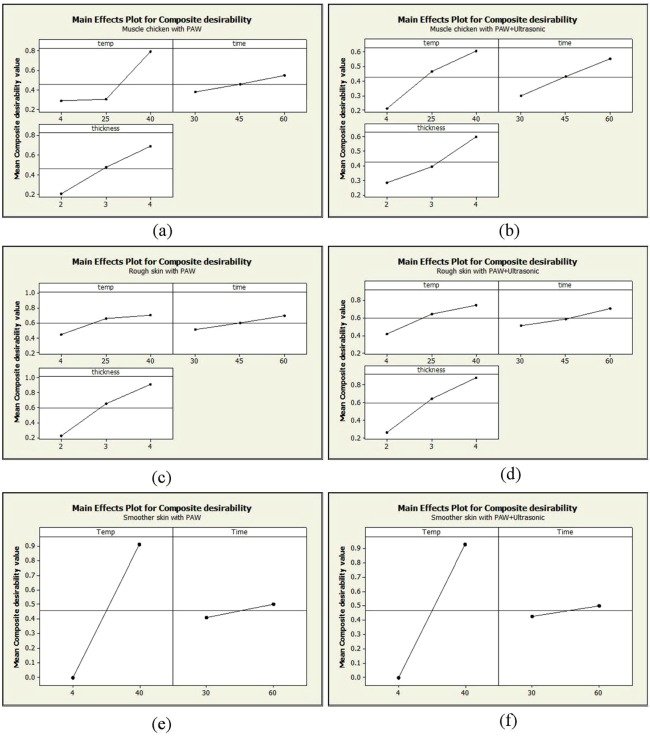
The main effect plot for composite desirability (**a**) muscle chicken meat part treated with PAW (**b**) muscle chicken meat part treated with PAW–ultrasound (**c**) rough skin chicken part treated with PAW (**d**) rough skin chicken part treated with PAW–ultrasound (**e**) smooth skin chicken part treated with PAW (**f**) smooth skin chicken part treated with PAW–ultrasound.

**Step 4:** In this step, ANOVA was performed to investigate the significance and influence of each parameter (Table 2). It indicated there was no significant difference in the factors between the chicken muscle and smooth skin (*p* > 0.05). However, rough chicken skin had significantly different skin thickness (*p* < 0.05). The influence of the parameters is represented as %C. For chicken muscle (PAW_%C_ = 54%, PAW–ultrasound_%C_ = 45%) and smooth skin (PAW_%C_ = 98%, PAW–ultrasound_%C_ = 99%), the temperature was the most influential parameter affecting the bacterial reduction. For rough skin, the skin thickness had the most influence on the bacterial reduction (PAW_%C_ = 80%, PAW–ultrasound_%C_ = 70%). The main effect plot showed a positive relationship among all the parameters with the responses, as shown in Fig. 3.

**Step 5:** The predicted value of the response was calculated based on the optimum level of the parameters. Table 1 provides the optimal condition for treating chicken muscle, rough skin, and smooth skin. For chicken muscle, the optimal condition was 40 °C, 60 min, and 4-mm thickness (A3B3C3), which presented an improved desirability value of 0.6484 (PAW) and 0.4763 (PAW–ultrasound). When applying the optimal condition to confirm the experiment, PAW and PAW–ultrasound reduced *E. coli* and *S. aureus* in chicken muscle by about 0.46 and 0.30, and 1.33 and 0.83 log CFU/ml, respectively. For rough skin, the optimal condition was also 40 °C, 60 min, and 4-mm thickness (A3B3C3), which presented improved desirability values of 0.5130 (PAW) and 0.5307 (PAW–ultrasound). The confirmation PAW and PAW–ultrasound experiments reduced *E. coli* and *S. aureus* on rough skin by about 0.56 and 0.43, and 1.12 and 0.86 log CFU/ml, respectively. Moreover, the optimal condition of PAW for smooth skin was 40 °C, 60 min, and 1-mm thickness (A2B2), which presented improved desirability values of 0.5000 (PAW) and 0.5000 (PAW–ultrasound). The confirmation PAW and PAW–ultrasound experiments on smooth skin reduced *E. coli* and *S. aureus* by about 0.35 and 0.08, and 0.73 and 0.10 log CFU/ml, respectively.

The empirical models for *E. coli* and *S. aureus* reduction were developed by using a regression model. The adequacy of the model was analyzed by the correlation coefficient *R*^2^ and adjusted correlation coefficient adj-*R*^2^. The empirical model for PAW-treated chicken muscle is represented by Eqs. (15) and (16), with *R*^2^ = 96.8% and adj-*R*^2^ = 87.0%, and for PAW–ultrasound-treated chicken muscle, by Eqs. (17) and (18), with *R*^2^ = 92.04% and adj-*R*^2^ = 68.17%:

15 $$E.\,coli\,reduction\,(\log \,{\rm{CFU}}/{\rm{ml}})=-\,0.1151+0.0033\,{\rm{temp}}+0.0009\,{\rm{time}}+0.0596\,{\rm{thickness}}$$

16 $$S.\,aureus\,{\rm{reduction}}\,(\log \,{\rm{CFU}}/{\rm{ml}})=-\,0.1458+0.0026\,{\rm{temp}}+0.0015\,{\rm{time}}+0.0488\,{\rm{thickness}}$$

17 $$E.\,coli\,{\rm{reduction}}\,(\log \,{\rm{CFU}}/{\rm{ml}})=0.2536+0.0080\,{\rm{temp}}+0.0079\,{\rm{time}}+0.0640\,{\rm{thickness}}$$

18 $$S.\,aureus\,{\rm{reduction}}\,(\log \,{\rm{CFU}}/{\rm{ml}})=0.0290+0.0037\,{\rm{temp}}+0.0022\,{\rm{time}}+0.0815\,{\rm{thickness}}$$

The empirical model for PAW-treated rough skin is described by Eqs. (19) and (20), with *R*^2^ = 97.5% and adj-*R*^2^ = 90.0% and, similarly, for PAW–ultrasound-treated rough skin by Eqs. (21) and (22), with *R*^2^ = 97.6% and adj-*R*^2^ = 90.50%:

19 $$E.\,coli\,{\rm{reduction}}\,(\log \,{\rm{CFU}}/{\rm{ml}})=-\,0.1088+0.0028\,{\rm{temp}}+0.0020\,{\rm{time}}+0.1117\,{\rm{thickness}}$$

20 $$S.\,aureus\,{\rm{reduction}}\,(\log {\rm{CFU}}/{\rm{ml}})=-\,0.1167+0.0015\,{\rm{temp}}+0.0018\,{\rm{time}}+0.0967\,{\rm{thickness}}$$

21 $$E.\,coli\,{\rm{reduction}}\,(\log \,{\rm{CFU}}/{\rm{ml}})=0.3845+0.0029\,{\rm{temp}}+0.0024\,{\rm{time}}+0.1083\,{\rm{thickness}}$$

22 $$S.\,aureus\,{\rm{reduction}}\,(\log \,{\rm{CFU}}/{\rm{ml}})=0.0925+0.0037\,{\rm{temp}}+0.0021\,{\rm{time}}+0.1133\,{\rm{thickness}}$$

The empirical model for PAW-treated smooth, thin skin is given by Eqs. (23) and (24), with *R*^2^ = 99.02% and adj-R^2^ = 97.06%, and, similarly, for PAW–ultrasound-treated smooth, thin skin as Eqs. (25) and (26), with *R*^2^ = 99.4% and adj-*R*^2^ = 98.1%:

23 $$E.\,coli\,{\rm{reduction}}\,(\log \,{\rm{CFU}}/{\rm{ml}})=0.0105+0.0072\,{\rm{temp}}+0.0008\,{\rm{time}}$$

24 $$S.\,aureus\,{\rm{reduction}}\,(\log \,{\rm{CFU}}/{\rm{ml}})=0.0379+0.0008\,{\rm{temp}}+0.0001\,{\rm{time}}$$

25 $$E.\,coli\,{\rm{reduction}}\,(\log \,{\rm{CFU}}/{\rm{ml}})=0.0744+0.0139\,{\rm{temp}}+0.0017\,{\rm{time}}$$

26 $$S.\,aureus\,{\rm{reduction}}\,(\log \,{\rm{CFU}}/{\rm{ml}})=0.0506+0.0011\,{\rm{temp}}$$

According to Taguchi experimental design, the regression Eqs. (15) to (26) derived from this study can be mathematically predicted the responses for reproduction of further study. The prediction of responses will be based on the changing of significant factor and the coefficient of the equation. Additional predicted value was shown as surface plot graph models in the supplementary. For instance, when the parameters of Eq. (18) are at the lower conditions (Temp = 4 °C, Time = 30 min, Thickness = 2 mm), the predicted value of *S. aureus* reduction will be 0.27 log CFU/ml. On the other hand, if the experimental parameters are changed into upper conditions (Temp = 40 °C, Time = 60 min, Thickness = 4 mm), the predicted value of *S. aureus* reduction will become 0.63 log CFU/ml. Both of these predicted values were insignificantly different to the experiment values which are 0.31 and 0.66 log CFU/ml, respectively.

## Scanning Electron Microscope (SEM)

**Figure 4:**
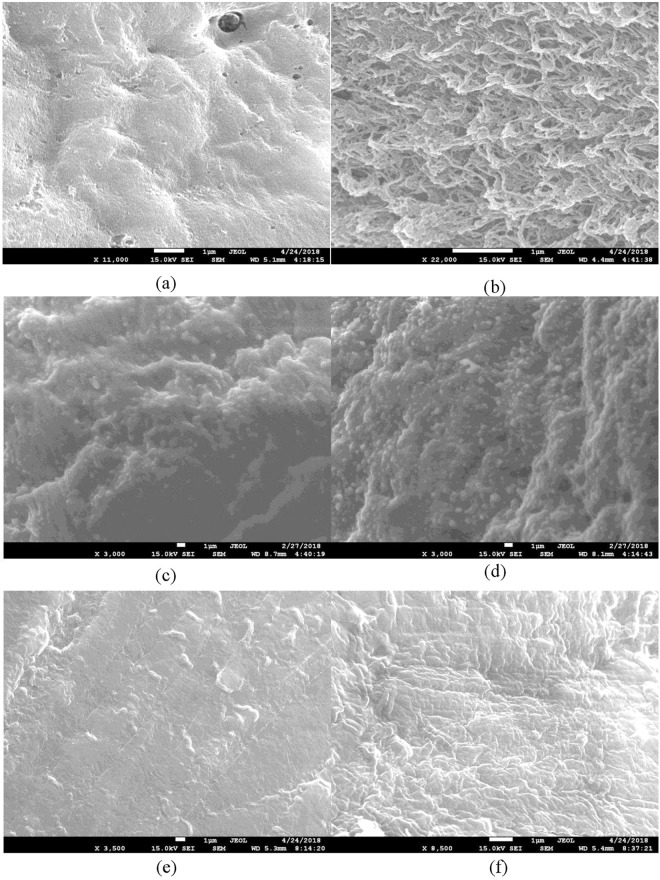
The SEM of (**a**) Muscle chicken soak with DI water (**b**) Muscle chicken soak with PAW–ultrasound (**c**) Rough skin chicken soak with DI water (**d**) Rough skin chicken soak with PAW–ultrasound (**e**) Smooth skin chicken soak with DI water (**f**) Smooth skin chicken soak with PAW–ultrasound.

Figure 4 displays the topography of the different types of chicken meat treated by DI water and PAW–ultrasound, as visualized by SEM. A comparison between the surface of chicken muscle treated with DI water only (Fig. 3a) and PAW–ultrasound treatment (Fig. 3b) showed the surface became more porous with ultrasound, which allowed PAW to penetrate the bacteria. However, the surface of rough and smooth skin was not different between the control (DI water) and PAW–ultrasound because of the denser texture and greater flexibility of the skin than muscle.

**Figure 5:**
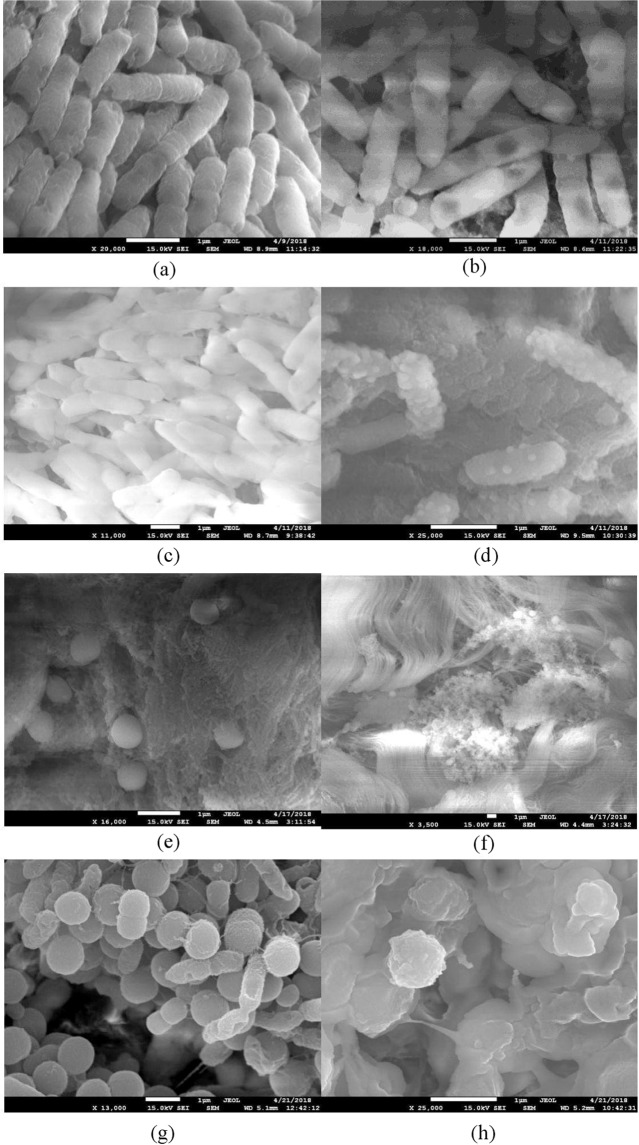
The SEM of *E. coli* and *S. aureus* topography on chicken (**a**) *E. coli* shape on muscle wash with DI water (**b**) *E. coli* shape on muscle wash with PAW–ultrasound (**c**) *E. coli* shape on skin wash with DI water (**d**) *E. coli* shape on skin wash with PAW–ultrasound (**e**) *S. aureus* shape on muscle wash with DI water (**f**) *S. aureus* shape on muscle wash with PAW–ultrasound (**g**) *S. aureus* shape on skin wash with DI water (**h**) *S. aureus* shape on skin wash with PAW–ultrasound.

Figure 5 shows the morphology of *E. coli* and *S. aureus* on muscle and skin before and after PAW treatment. In chicken muscle, both bacteria remained as a group attached to the fibrin (Fig. 5a,c,g). Additionally, some *S. aureus* on chicken muscle stayed separated and embedded in the tissue (Fig. 5e). After PAW treatment, the membranes of *E. coli* were broken, but the bacteria retained its rod shape (Fig. 5b). Severe physical damage was induced by PAW treatment on *S. aureus* (Fig. 5f). On the contrary, the SEM observations of *E. coli* and *S. aureus* on chicken skin indicated that both bacteria retained their shape, but the surface morphology was altered, and bubble-like protrusions appeared on the bacterial membrane (Fig. 5d,h).

## Quality analysis

### Color measurement

The average and standard deviation of L*, a*, b*, and Δ*E* are shown in Table 3. The perceivable color change can be categorized as ‘very distinct’ (Δ*E* > 3), ‘distinct’ (1.5 < Δ*E* < 3), and ‘small differences’ (Δ*E* < 1.5)^21^. In this study, the Δ*E* values of chicken muscle and rough chicken skin were both found to be ‘very distinct’ when treated by ultrasound and PAW–ultrasound, and ‘distinct’ for PAW treatment, whereas, the Δ*E* values of smooth chicken skin were ‘very distinct’ under all conditions. When Δ*E* > 3, the human eye should be able to detect the color differences distinctly^22^. However, the sensory panel did not notice differences in the color of chicken muscle due to ultrasound and PAW–ultrasound.

**Table 3:** Color measurement, hardness measurement, protein and lipid measurement of muscle chicken meat, rough skin chicken, and smooth skin chicken after treated by different technique at 40 °C.

Type	Treatment	L*	a*	b*	ΔE	Hardness (N)	Protein (%)	Lipid (%)
Muscle	Water	59.82 ± 1.27a	1.57 ± 0.61a	4.61 ± 2.05a		90.97 ± 35.45a	23.45 ± 0.65a	7.53 ± 0.17a
	PAW	62.02 ± 1.83a	0.86 ± 0.58a	4.77 ± 0.99a	2.89	90.43 ± 6.19a	22.20 ± 1.15a	7.62 ± 0.41a
	Ultrasound	62.90 ± 3.72a	1.26 ± 1.07a	4.70 ± 1.61a	5.55	84.18 ± 11.70a	24.24 ± 0.75a	7.10 ± 0.92a
	PAW-ultrasound	63.74 ± 5.56a	0.77 ± 1.58a	4.63 ± 3.28a	4.85	88.71 ± 12.97a	22.91 ± 0.03a	7.54 ± 0.47a
Rough skin	Water	75.01 ± 0.18a	−1.14 ± 0.15a	8.26 ± 1.12a		29.81 ± 2.90a	6.59 ± 0.62a	72.3 ± 2.96a
	PAW	75.54 ± 0.68a	−0.59 ± 0.47 a	9.15 ± 1.31a	2.10	27.39 ± 1.93a	6.36 ± 0.14a	70.34 ± 3.19a
	Ultrasound	73.92 ± 1.42a	−1.10 ± 0.75a	4.39 ± 0.48b	4.32	30.58 ± 2.95a	6.59 ± 0.27a	71.82 ± 0.83a
	PAW-ultrasound	74.61 ± 0.59a	−0.30 ± 0.60a	8.68 ± 1.37a	2.02	30.62 ± 3.85a	6.77 ± 0.22a	71.86 ± 3.43a
Smooth skin	Water	74.65 ± 0.59a	−0.6 ± 0.99a	5.22 ± 2.29a		2.52 ± 0.31a	8.73 ± 1.08a	69.41 ± 1.12a
	PAW	74.55 ± 0.36a	−0.58 ± 0.38b	8.66 ± 0.09a	3.52	2.67 ± 0.16a	11.96 ± 0.53a	69.81 ± 0.82a
	Ultrasound	74.67 ± 0.47a	−1.76 ± 0.21a	5.57 ± 3.67a	4.01	2.98 ± 0.21a	8.83 ± 0.73a	70.11 ± 0.70a
	PAW-ultrasound	74.88 ± 0.32a	−0.77 ± 0.26a	8.87 ± 1.17a	4.06	3.03 ± 0.76a	9.52 ± 0.84a	68.76 ± 0.95a

### Hardness measurement

The hardness of chicken muscle, rough skin, and smooth skin did not change significantly due to the ultrasound, PAW, and PAW–ultrasound at 40 °C for 60 min (Table 3). This result might have been because of the low amount of energy dissipated to the system by the ultrasonic bath^23^. According to Lyng *et al*.^24^, the intensity of ultrasound was insufficient to change the muscle properties and may have been too low to alter the texture due to the high content of connective tissue in chicken meat.

### Protein and lipid measurement

There were no significant differences in the protein and lipid contents between the non-treated chicken meat and chicken meat treated by PAW–ultrasound. In general, non-thermal plasma can decrease the protein content of a sample by heat, charged particles, the intense electric field, UV photons, and neutral reactive species. However, all five factors are presented only in gas discharge^25^.

The chicken muscle has less lipid than the chicken skin, and the rough chicken skin has more lipid than the smooth chicken skin. Samples treated with PAW–ultrasound were not significantly different from their untreated counterparts. It is likely that because of the lower lipid amount in chicken muscle than skin, the most damage induced by the ROS in PAW and ultrasound was due to the interaction of the ROS with the bacterial cell wall rather than the chicken skin.

### Sensory evaluation

For chicken muscle, rough skin and smooth skin, all the tested sensory properties were not significantly (*p* > 0.05) irrespective of the treatment as compared with the untreated controls. However, the specialist sensory panel scored the overall properties of chicken muscle, rough skin, and smooth skin when treated by ultrasound (6.67 ± 0.52, 6.00 ± 1.20, and 6.25 ± 1.04, respectively), PAW (6.83 ± 0.41, 6.63 ± 0.74, and 6.50 ± 0.76, respectively), and PAW–ultrasound (6.50 ± 0.55, 6.00 ± 0.76, and 6.25 ± 0.89, respectively) above category 5 (neither like nor dislike) (Table 4).

**Table 4:** Sensory evaluation from 10 specialist using 9-point hedonic of muscle chicken meat, rough skin chicken, and smooth skin chicken after treated by different technique at 40 °C.

Type	Treatment	Appearance	Color	Texture	Acceptability
Muscle	Water	7.00 ± 0.76a	6.83 ± 0.75a	6.50 ± 0.93a	6.50 ± 0.55a
	PAW	6.50 ± 0.53a	5.67 ± 1.03a	6.38 ± 0.92a	6.83 ± 0.41a
	Ultrasound	6.13 ± 0.99a	5.66 ± 1.63a	6.00 ± 0.76a	6.67 ± 0.52a
	PAW-ultrasound	6.00 ± 1.07a	5.83 ± 1.17a	6.00 ± 0.93a	6.50 ± 0.55a
Rough skin	Water	7.00 ± 0.93a	6.25 ± 0.46a	5.75 ± 1.04a	6.63 ± 0.92a
	PAW	7.00 ± 0.93a	5.75 ± 1.16a	6.00 ± 0.76a	6.63 ± 0.74a
	Ultrasound	5.88 ± 1.73a	5.75 ± 0.71a	6.00 ± 0.76a	6.00 ± 1.20a
	PAW-ultrasound	6.25 ± 1.28a	5.63 ± 1.06a	5.88 ± 0.99a	6.00 ± 0.76a
Smooth skin	Water	7.00 ± 0.93a	6.63 ± 0.74a	6.38 ± 0.52a	6.75 ± 1.04a
	PAW	6.50 ± 1.20a	6.13 ± 0.83a	6.25 ± 0.71a	6.50 ± 0.76a
	Ultrasound	6.25 ± 1.39a	6.00 ± 0.93a	6.25 ± 0.71a	6.25 ± 1.04a
	PAW-ultrasound	6.63 ± 0.92a	6.00 ± 1.07a	6.25 ± 0.71a	6.25 ± 0.89a

Values are given as mean ± SD (n = 10). Means in the same column followed by the same letter are not significantly (P > 0.05) different by Turkey’s test with error rate = 5.

## Discussion

The key inactivation agents of non-thermal plasma are RONS. This research focuses on the ROS in PAW, notably the long-lived ROS, such as H_2_O_2_, which is responsible for the bacterial reduction. The bacteria inactivation mechanism can be explained by the following four steps. In the first step, the underwater Ar plasma jet produces high amounts of ROS. In the second step ROS, especially •OH, initiate lipid peroxidation of the lipid bilayer in the bacterial cell membrane, leading to changes in cell permeability and depolarization of the cell membrane. In the third step, ROS move through transient pores and induce oxidative stress in the cell, to increase intracellular ROS. In the forth step, intracellular ROS react with proteins, lipids, and carbohydrates, leading to alteration of molecule structures and chemical bonds. However, Zhang *et al*.^24^ reported a reduction in ROS when adding organic matter to PAW. Therefore, excess intercellular ROS and low-pH induced redox reactions and disrupted pH homeostasis in the cell, causing cell death^26^. In turn, the ultrasound increased the penetration rate of PAW through the samples.

The bacteria morphology on chicken muscle exhibited severe physical damage as compared with the bacteria on the skin, due to more organic matter in the skin than muscle. According to Almeida *et al*.^27^, chicken has a high content of polyunsaturated fatty acids (PUFA) of about 21.3 ± 3.5%. A high concentration of omega-3 and omega-6 PUFAs can inhibit ROS and RNS formation^28^. The ROS target C–H bonds, especially double bonds because of the lower energy required to abstract a hydrogen atom (272 kJ/mol) than another C–H bond (422 kJ/mol)^29^. Therefore, the efficiency of ROS to inhibit bacteria present on chicken skin was decreased, indicated by the protrusion of the bacterial membrane and not the disintegration of bacteria evidenced on the chicken muscle.

The ultrasound process caused membrane disruption, which released unsaturated lipids that reacted with free radicals in PAW. This process is called lipid oxidation. Most of lipid oxidation reaction were found in muscle foods which caused the change of color, odor, taste, and shelf life. There are many studies that found lipid oxidation such as fresh pork and beef treated with dielectric barrier discharge plasma^30^, pork loin treated with helium and oxygen plasma^31^, and also ground pork treated with plasma jet^32^.

In this study, our technique dramatically inactivated the number of *E. coli* 2.12 and 1.37 log CFU/ml at the initial microbial 10^5^ and 10^6^ CFU/ml, respectively, in the case of real situation at low concentration of microorganisms contaminated in foods. Those results agreed to the reports at the difference of initial microbial effects with the amount of bacterial inoculum with the levels of 10^2^, 10^3^, 10^4^ CFU/ml of *S. enterica* and were reduced the survival of microorganism to 92.5%, 87.0%, 63.5% on chicken breast and 62.5%, 36.0%, 32.8% on chicken skin, respectively^6^.

## Materials and Methods

### Sample preparation

Fresh, raw whole chickens were purchased at a local market and transported immediately to the laboratory. Each chicken was separated to obtain two boneless breasts with skin and kept at 4 °C overnight before use. The skin and muscle were separated by using scalpel and forceps, then placed in a Petri dish. Both parts were soaked in sterilized deionized (DI) water for 5 min and dried for 5 min before use. Three different types of samples were investigated; chicken muscle (meat), rough skin, and smooth, thin skin. The samples were punched using a sterile cork-borer to a diameter of 1 cm. The muscle was 2 mm in thickness, and the skin thickness was 2, 3, and 4 mm for rough skin, and 1 mm for smooth, thin skin. All samples were kept in 15-ml centrifuge tubes.

Due to the different weight and thickness of chicken muscle and skin, the bacterial suspension was deposited differently. The muscle and rough skin at 2, 3, and 4 mm were inoculated with 50 µl of the bacterial suspension, and the smooth, thin skin (1 mm) was inoculated with 20 µl of the suspension. After inoculation, the tissue samples were kept in laminar flow at room temperature for 1 h to allow attachment following the method of Noriega *et al*.^5^. The experiment was triplicate.

### Bacteria culture and sample inoculation

*Escherichia coli* K12 (KCTC1116; Korea Research Institute of Bioscience and Biotechnology) and *Staphylococcus aureus* type strain ATCC12600 (KCCM12103; Korea Culture Center of Microorganisms) were used in this research. A stock culture was maintained in sterile glycerol solution at −80 °C. Fresh cultures of *E. coli* and *S. aureus* were cultivated in nutrient broth by shaking at 200 rpm, 37 °C for 24 h at the stationary phase, and diluted to a final concentration of approximately 10^7^–10^8^ CFU/ml, which was used for the inoculation of chicken meat and skin.

### Plasma device, PAW generation, and ultrasound treatment

A volume of 20 ml of DI water (Milli-Q® Direct Water Purification System, Merck KGaA, Darmstadt, Germany) was used in the experiment (Fig. 1). The single-electrode non-thermal atmospheric pressure underwater plasma jet with a direct current (DC) positive fly-back transformer was used to generate PAW in this experiment^33,34^. The device consists of a copper wire as the single electrode cover with a quartz tube and stainless-steel ground. The reactor is connected to a 1.5-kHz square high-voltage source with a 6.8-kV peak-to-peak voltage. The device is set up to discharge PAW beneath the water surface at a distance of 5 mm between the electrode and ground. This setup is the optimized condition of the device, which discharges PAW using Ar gas at a flow rate of 3 slm for 6.5 min.

The PAW volume was based on the recommendation of one of the poultry industries in Thailand. Therefore, the tissue sample at 1 (0.08 ± 0.01 g), 2 (0.15 ± 0.03 g), 3 (0.22 ± 0.03 g), and 4 mm (0.30 ± 0.03 g) used PAW volumes of 309, 580, 846, and 1160 µl, respectively. The tissue samples were soaked in PAW and ultrasonicated at 4, 25, and 40 °C for 30, 45, and 60 min. The ultrasonic benchtop device (Powersonic 603, Kleentek, Queensland, Australia) operated at an ultrasonic frequency of 40 Hz and an output power of 220 W.

### Physiochemical properties

The OES (HR4000CG-UV-NIR Spectrometer, Wonwoo System Co., Ltd., Seoul, Korea) was used to identify the reactive species during PAW treatment. The pH, ORP, EC, H_2_O_2_, and •OH were measured after PAW and ultrasound. The values of pH, ORP, and EC were measured using a pH spear (Eutech, Korea), an ORP tester (HI98201, Hanna Instruments, Romania), and an EC tester (HI98393 DIST® 3, Hanna Instruments), respectively. The H_2_O_2_ concentration was quantified using a QuantiChrom™ Peroxide Assay Kit, and the •OH concentration was determined by fluorescence spectroscopy (Synergy HT, BioTek Instruments Korea Ltd)^35^.

### Statistical analysis

#### One-way analysis of variance (ANOVA)

The physicochemical properties (pH, ORP value, EC value, •OH concentration, and H_2_O_2_ concentration), bacterial log reduction values, and quality analyses (color, hardness, proximate analysis, and sensory evaluation) of DI water, and DI water with ultrasound, PAW, and PAW–ultrasound were subjected to one-way ANOVA, followed by Tukey’s test at a 5% significance level, using Minitab 16 statistical software, Minitab, LLC, PA, USA.

#### Taguchi experimental plan

The optimization of the meat conditions was investigated by the desirability function embedded in Taguchi analysis using Minitab 16 software to construct and analyze the experiments. As there are three factors for each chicken part; temperature, thickness, and treatment time, an L9 Taguchi orthogonal design was used to investigate the experimental parameters of chicken muscle and rough skin. The design summary is shown in Table 5. However, for the smooth skin, with a single thickness of 1 mm, a 2-factor, 2-level (L4) Taguchi experimental design was utilized.

**Table 5:** Experiment layout using an L9 orthogonal array and corresponding results each chicken type with PAW and PAW-ultrasound.

Temp (°C)	Time (min)	Thickness (mm)	Muscle chicken				Rough skin			
			PAW		PAW-ultrasound		PAW		PAW-ultrasound	
			*E. coli* (log CFU/ml)	*S. aureus* (log CFU/ml)	*E. coli* (log CFU/ml)	*S. aureus* (log CFU/ml)	*E. coli* (log CFU/ml)	*S. aureus* (log CFU/ml)	*E. coli* (log CFU/ml)	*S. aureus* (log CFU/ml)
4	30	2	0.08 ± 0.01	0.02 ± 0.00	0.65 ± 0.04	0.31 ± 0.03	0.20 ± 0.03	0.11 ± 0.02	0.69 ± 0.08	0.38 ± 0.02
4	45	3	0.15 ± 0.03	0.10 ± 0.00	0.84 ± 0.03	0.32 ± 0.03	0.34 ± 0.03	0.30 ± 0.02	0.85 ± 0.06	0.58 ± 0.04
4	60	4	0.18 ± 0.06	0.16 ± 0.02	1.12 ± 0.02	0.46 ± 0.07	0.43 ± 0.00	0.35 ± 0.02	0.94 ± 0.01	0.66 ± 0.03
25	30	3	0.10 ± 0.02	0.08 ± 0.01	0.91 ± 0.03	0.42 ± 0.06	0.35 ± 0.07	0.29 ± 0.01	0.85 ± 0.02	0.61 ± 0.03
25	45	4	0.20 ± 0.02	0.16 ± 0.03	0.97 ± 0.03	0.69 ± 0.13	0.52 ± 0.00	0.39 ± 0.03	1.02 ± 0.01	0.71 ± 0.01
25	60	2	0.11 ± 0.01	0.07 ± 0.06	0.86 ± 0.02	0.45 ± 0.24	0.32 ± 0.02	0.24 ± 0.01	0.84 ± 0.11	0.56 ± 0.02
40	30	4	0.33 ± 0.03	0.21 ± 0.02	1.01 ± 0.03	0.52 ± 0.01	0.51 ± 0.00	0.38 ± 0.01	1.01 ± 0.02	0.77 ± 0.01
40	45	2	0.16 ± 0.01	0.15 ± 0.02	1.21 ± 0.03	0.42 ± 0.02	0.27 ± 0.02	0.19 ± 0.02	0.79 ± 0.06	0.52 ± 0.04
40	60	3	0.30 ± 0.02	0.22 ± 0.05	1.30 ± 0.02	0.53 ± 0.07	0.49 ± 0.03	0.35 ± 0.06	0.99 ± 0.01	0.73 ± 0.05
			**Smooth skin**							
			**PAW**		**PAW-ultrasound**		**PAW**		**PAW-ultrasound**	
			***E. coli*** **(log CFU/ml)**		***S. aureus*** **(log CFU/ml)**		***E. coli*** **(log CFU/ml)**		***S. aureus*** **(log CFU/ml)**	
4	30	1	0.07 ± 0.00		0.05 ± 0.01		0.07 ± 0.00		0.05 ± 0.00	
4	60	1	0.09 ± 0.00		0.04 ± 0.00		0.09 ± 0.01		0.04 ± 0.00	
40	30	1	0.32 ± 0.03		0.07 ± 0.01		0.32 ± 0.02		0.07 ± 0.01	
40	60	1	0.35 ± 0.01		0.08 ± 0.00		0.35 ± 0.03		0.08 ± 0.01	

#### Desirability function approach (DFA)

To obtain the best results of all responses simultaneously, the DFA^36^ described by Derringer and Suich^37^ was used. The desirability function is strongly suggested for optimizing the multiple response problem^38^. It converts each response into an individual desirability function (*d*_*i*_) that has values between 0 to 1 (least to most desirable, respectively). In this research, the factors are temperature, time, and thickness. The responses are *E. coli* and *S. aureus* log CFU/ml reduction. The factor setting with nominal total desirability is the optimal parameter conditions. Therefore, the DFA optimization has five steps, as follows:

**Step 1**: Calculate the *d*_*i*_^35^. In this case, the maximal bacterial reduction of both *E. coli* and *S. aureus* were investigated. The equation of individual desirability of the-larger-the-better is shown in Eq. (15):

27 $${d}_{i}=\left\{\begin{array}{ll}0, & \hat{y}\le {y}_{min}\\ {\left(\frac{\hat{y}-{y}_{min}}{{y}_{max}-{y}_{min}}\right)}^{r}, & {y}_{min}\le \hat{y}\le {y}_{max},\,r\ge 0\\ 1, & \hat{y}\ge {y}_{max}\end{array}\right.$$

where *y*_*min*_ and *y*_*max*_ represent the lower and upper tolerance limit of $\hat{y}$, respectively; and *r* indicate the weights that depend on the user.

**Step 2:** Evaluate the composite desirability (*d*_*G*_) by the following equation:

28 $${d}_{G}={({d}_{1}^{w1}{d}_{2}^{w2}{d}_{3}^{w3}\ldots \mathrm{.}.{d}_{n}^{wn})}^{1/W}$$

where *d*_*i*_ is the individual desirability of the property *Y*_*i*_; *w*_*i*_ is the weight of *Y*_*i*_, and *W* is the sum of the individual weights.

**Step 3**: Optimize the *d*_*G*_ value. The highest *d*_*G*_ indicates better product quality.

**Step 4**: Investigate the significance of each parameter by using ANOVA.

**Step 5**: Calculate the prediction of the optimal condition and validate the results.

### Microbiological analysis

#### Microbiological counts

The chicken sample was transferred to sterilized bags containing 0.85% saline solution at 10 times the volume of the sample. The tissue samples were crushed by hand, applying the same force for 2 min, and all samples were considered to be evenly mixed. For the enumeration of *E. coli* and *S. aureus*, aliquots (100 µL) of cell suspension or 10-fold serial dilutions were spread onto nutrient agar (NA) plates and the plates incubated at 35 °C for 20–24 h.

#### Scanning electron microscopy (SEM)

Skin and muscle samples were fixed in paraformaldehyde and glutaraldehyde for 2.5 h before SEM analysis. Each sample was washed twice in 10 × PBS (15 min each time) and then dehydrated with increasing ethanol concentrations (30, 50, 70, 90, and 100% v/v) for 15 min each, and dried by hexamethyldisilazane for 10 min twice. All samples were mounted on brass stubs via double-sided carbon sticky-tape, sputtered with platinum in a vacuum evaporator, and visualized under a field-emission SEM microscope (Jeol JSM-7001F, JEOL Ltd., Tokyo, Japan).

### Quality of chicken meat analysis

#### Color measurement

The surface color of the muscle, rough skin, and smooth skin was measured before and after treatment by using a Minolta CR-300 colorimeter (Minolta Co., Ltd, Osaka, Japan) to record the CIE L* (lightness), a* (redness/greenness), and b* (yellowness/blueness) coordinates. Δ*E* is an indicator of the overall chicken sample color changes^39^, which is calculated as:

29 $$\Delta E=\sqrt{{(\Delta {L}^{\ast })}^{2}+{(\Delta {a}^{\ast })}^{2}+{(\Delta {b}^{\ast })}^{2}}$$

where ΔL*, Δa*, and Δb* are the differences in sample color before and after treatment.

#### Hardness measurement

The hardness of chicken samples before and after treatment was analyzed using a TA.XTplus texture analyzer (Texture Technologies Corp., Stable Micro Systems, Ltd., Godalming, UK) with a strain of 70%, trigger force of 10 g, and 3.5-cm-diameter flat-faced cylindrical probe.

#### Protein and lipid measurement

The samples were analyzed for protein content using a combustion technique (Leco FP-528, USA) and adopting Jones’ factor for meat (6.25) to convert the nitrogen content to protein content (Eq. (30)). Crude lipid content was determined by Soxhlet extraction with petroleum ether (Soxtec^TM^ 8000 Extraction Unit, Foss Company, Denmark) and applying Eq. (31) to calculate the results.

30 $${\rm{Protein}}( \% )={\rm{Nitrogen}}( \% )\times 6.25$$

31 $${\rm{Lipid}}( \% )=[{\rm{Weight}}\,{\rm{after}}\,{\rm{extraction}}\,({\rm{g}})-{\rm{Weight}}\,{\rm{before}}\,{\rm{extraction}}\,({\rm{g}})]\times 100/{\rm{Sample}}\,({\rm{g}})$$

#### Sensory evaluation

The sensory attributes of appearance, color, texture, and acceptability of the product were evaluated using a 9-point hedonic scale (9 = like extremely; 1 = dislike extremely). The chicken muscle, rough skin, and smooth skin (untreated, ultrasound-treated, PAW-treated, and PAW–ultrasound-treated) were sensory evaluated by a 10-member panel consisting of staff members from the poultry industry who were previously experienced in quality evaluation.

## Conclusions

The desirability function with Taguchi can be used for optimizing the bacteria reduction by using PAW and PAW–ultrasound with different types of chicken meat. The SEM results showed that ultrasound could destroy the chicken muscle cells, facilitating the entry of ROS from PAW into the bacteria. Moreover, the increasing temperature could lead to a weakened cell wall of the bacteria and leave the cell membrane less protected from other treatment. The muscle meat at 4 mm thickness, soaked in PAW at 40 °C for 60 min reduced the bacterial load of *E. coli* by 0.46 log CFU/ml and *S. aureus* by 0.30 log CFU/ml. Under the same condition, PAW–ultrasound reduced the *E. coli* count by 1.33 log CFU/ml and *S. aureus* by 0.83 log CFU/ml. For the rough chicken skin, the thickness of the skin was the most important factor affecting the bacteria reduction (*p* < 0.05) by both PAW and PAW–ultrasound. The optimal condition, which was the same as that of the chicken muscle, reduced *E. coli* by 0.56 and 1.12 log CFU/ml and *S. aureus* by 0.43 and 0.86 log CFU/ml when treated with PAW and PAW–ultrasound, respectively. For smooth chicken skin, the best treatment (40 °C for 60 min) reduced *E. coli* by 0.35 and 0.08 log CFU/ml, and *S. aureus* by 0.73 and 0.10 log CFU/ml, for PAW and PAW–ultrasound, respectively. The main effect factor plot showed temperature had more influence on the physicochemical properties of PAW than the activated time (*p* < 0.10). The hardness, protein, and lipid measurements of the treated samples were comparable to those of their untreated counterparts. This study proved that the synergistic effect of combined PAW and ultrasound was more effective for reducing the bacterial load than either technique alone, which was significant with natural contamination.

## Supplementary information

Table SS1, Table SS2, Figure SS1.
