# Supplementary material for: Chemical-free and synergistic interaction of ultrasound combined with plasma-activated water (PAW) to enhance microbial inactivation in chicken meat and skin
Source: Sci Rep. 2020 Jan 31;10:1559. doi: 10.1038/s41598-020-58199-w (PMC6994601; doi:10.1038/s41598-020-58199-w)
Supplement: Supplementary file 1 — Table SS1, Table SS2, Figure SS1. [file 41598_2020_58199_MOESM1_ESM.docx]

**Chemical-free and synergistic interaction of ultrasound combined with plasma-activated water (PAW) to enhance microbial inactivation in chicken meat and skin**

Keywords: Plasma Activated Water (PAW), Ultrasound, Taguchi, Desirability function, Chicken meat, Bacteria

Tanitta Royintarat^1^, Eun Ha Choi^2^, Dheerawan Boonyawan^3^ Phisit Seesuriyachan^1,4,*^ and Wassanai Wattanutchariya^1,*^

^1^Advanced Manufacturing Technology Research Center (AMTech), Department of Industrial Engineering, Faculty of Engineering, Chiang Mai University, Chiang Mai, Thailand 50200

^2^Plasma Bioscience Research Center, Kwangwoon University, Seoul, Korea

^3^Department of Physics and Materials Science, Faculty of Science, Chiang Mai University, Chiang Mai, Thailand 50200

^4^Cluster of Agro Bio-Circular-Green Industry, Faculty of Agro-Industry, Chiang Mai University, Chiang Mai, Thailand 50200

^1^royintarat@hotmail.com; ^1,4,*^ phisit.s@cmu.ac.th; ^1,*^wassanai@eng.cmu.ac.th,

**Supplementary data**

**Table SS1** Design of Experiment (DOE) by Taguchi method for muscle chicken, roughness skin chicken and smoother skin chicken part are using PAW and PAW combine with ultrasound.

| **Factors (muscle and rough skin chicken)** | | | | **Range and label** | | | | | | | | |
| --- | --- | --- | --- | --- | --- | --- | --- | --- | --- | --- | --- | --- |
|  |  |  |  | **Low (1)** | | **Middle (2)** | | | | | **High (3)** | |
| Temperature (^o^C) | | | | 4 | | 25 | | | | | 40 | |
| Time (min) | | | | 30 | | 45 | | | | | 60 | |
| Thickness (mm) | | | | 2 | | 3 | | | | | 4 | |
| **No.** | **Temperature, ^o^C** | | | | **Time, min** | | | | **Thickness, mm** | | | |
|  | **Code** | | **Real** | | **Code** | | **Real** | | **Code** | | | **Real** |
| 1 | 1 | | 4 | | 1 | | 30 | | 1 | | | 2 |
| 2 | 1 | | 4 | | 2 | | 45 | | 2 | | | 3 |
| 3 | 1 | | 4 | | 3 | | 60 | | 3 | | | 4 |
| 4 | 2 | | 25 | | 1 | | 30 | | 2 | | | 3 |
| 5 | 2 | | 25 | | 2 | | 45 | | 3 | | | 4 |
| 6 | 2 | | 25 | | 3 | | 60 | | 1 | | | 2 |
| 7 | 3 | | 40 | | 1 | | 30 | | 3 | | | 4 |
| 8 | 3 | | 40 | | 2 | | 45 | | 1 | | | 2 |
| 9 | 3 | | 40 | | 3 | | 60 | | 2 | | | 3 |
| **Factors**  **(smooth skin chicken)** | | **Range and label** | | | | | | | | | | |
|  |  | **Low (1)** | | | | | | **High (2)** | | | | |
| Temperature (^o^C) | | 4 | | | | | | 40 | | | | |
| Time (min) | | 30 | | | | | | 60 | | | | |
| **No.** | **Temperature, ^o^C** | | | | | **Time, min** | | | | | | |
|  | **Code** | | | **Real** | | **Code** | | | | **Real** | | |
| 1 | 1 | | | 4 | | 1 | | | | 30 | | |
| 2 | 1 | | | 4 | | 2 | | | | 60 | | |
| 3 | 2 | | | 40 | | 1 | | | | 30 | | |
| 4 | 2 | | | 40 | | 2 | | | | 60 | | |

**Table SS2** Evaluated desirability function results of each chicken type with PAW and PAW-ultrasound.

| temp (^o^C) | time (min) | thickness (cm) | Composite desirability | | | | | |
| --- | --- | --- | --- | --- | --- | --- | --- | --- |
|  |  |  | Muscle chicken | | Rough skin | | Smooth skin | |
|  |  |  | PAW | PAW-ultrasound | PAW | PAW-ultrasound | PAW | PAW-ultrasound |
| 4 | 30 | 2 | 0.0000 | 0.0000 | 0.0000 | 0.0000 |  |  |
| 4 | 45 | 3 | 0.3347 | 0.0877 | 0.5449 | 0.4986 |  |  |
| 4 | 60 | 4 | 0.5220 | 0.5429 | 0.7849 | 0.7375 |  |  |
| 25 | 30 | 3 | 0.1549 | 0.3403 | 0.5489 | 0.5347 |  |  |
| 25 | 45 | 4 | 0.5797 | 0.7016 | 1.0000 | 0.9199 |  |  |
| 25 | 60 | 2 | 0.1678 | 0.3505 | 0.4173 | 0.4580 |  |  |
| 40 | 30 | 4 | 0.9747 | 0.5532 | 0.9665 | 0.9847 |  |  |
| 40 | 45 | 2 | 0.4561 | 0.4994 | 0.2500 | 0.3298 |  |  |
| 40 | 60 | 3 | 0.9381 | 0.7609 | 0.8814 | 0.9032 |  |  |
| 4 | 30 | 1 |  |  |  |  | 0.0000 | 0.0000 |
| 4 | 60 | 1 |  |  |  |  | 0.0000 | 0.0000 |
| 40 | 30 | 1 |  |  |  |  | 0.8183 | 0.8528 |
| 40 | 60 | 1 |  |  |  |  | 1.0000 | 1.0000 |
| Total mean value of the composite desirability | | | 0.4587 | 0.4263 | 0.5993 | 0.5963 | 0.4546 | 0.4632 |

(a) (b)****

(c) (d)

(e) (f)

(g) (h)

**** (i) (j)

(k) (l)

**Figure SS1** The prediction surface plot graph of chicken meat and skin at optimal condition (a) muscle (PAW) of *E.coli* (b) muscle (PAW) of *S.aureus* (c) muscle (PAW-ultrasound) of *E.coli* (d) muscle (PAW-ultrasound) of *S.aureus* (e) rough skin (PAW) of E.coli (f) rough skin (PAW) of *S.aureus* (g) rough skin (PAW-ultrasound) of *E.coli* (h) rough skin (PAW-ultrasound) of *S.aureus* (i) smooth skin (PAW) of *E.coli* (j) smooth skin (PAW) of *S.aureus* (k) smooth skin (PAW-ultrasound) of *E.coli* (l) smooth skin (PAW-ultrasound) of *S.aureus*
